# Management of Refractory Bleeding at the Duodenal Bulb Post‐Trauma Using OTSC Combined With Interventional Embolization: A Case Report

**DOI:** 10.1002/ccr3.70486

**Published:** 2025-05-05

**Authors:** Qing Chen, Shifeng Shao

**Affiliations:** ^1^ Department of ICU, Daping Hospital Army Medical University Chongqing China

**Keywords:** interventional embolization, over‐the‐scope clip, post‐trauma, stress ulcer

## Abstract

Multiple traumas can lead to acute gastrointestinal mucosal erosion or ulcers, typically presenting as upper gastrointestinal bleeding, while perforation occurs rarely. Rebleeding poses a serious threat to life. Standard treatments include endoscopic electrocoagulation, tissue adhesives, and titanium clips. We report a case of a 39‐year‐old woman with post‐traumatic stress ulcer bleeding successfully managed using a combination of over‐the‐scope clip (OTSC) placement and interventional embolization. This case demonstrates the effectiveness of OTSC combined with embolization in controlling post‐traumatic ulcer bleeding and preventing recurrence.

**Summary**

- This paper explores the use of combined Over‐the‐Scope Clip (OTSC) placement and interventional embolization in treating duodenal stress ulcer bleeding.
- The approach offers a promising option for managing cases that do not respond to conventional treatment.

## 1 Introduction

Stress ulcer bleeding occurs in about 4.7% of critically ill patients, with clinically significant cases accounting for 2.8% [1]. Severe trauma is a key risk factor, and once bleeding begins, the mortality rate can rise to 50%–80% [2]. Although endoscopic hemostasis has primarily replaced surgery as the preferred treatment, failure still occurs in 6%–28% of cases [3]. In the reported case, the patient experienced repeated upper gastrointestinal bleeding that did not respond to multiple endoscopic attempts. Surgical intervention posed high risks with low benefits. While previous studies have shown that over‐the‐scope clip (OTSC) is effective for non‐variceal bleeding, this case highlights the successful use of OTSC in combination with interventional embolization as a treatment strategy [4, 5].

## 2 Case History/Examination

**FIGURE 1:**
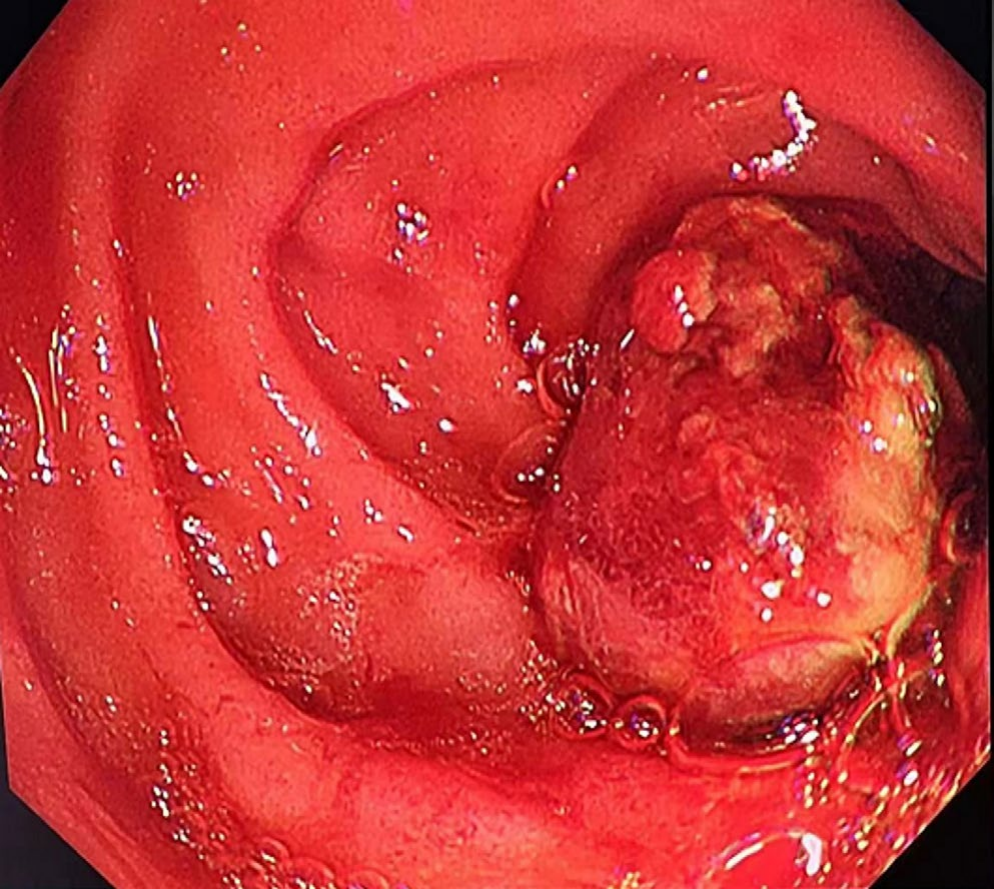
Endoscopic view pre‐clipping. Duodenoscopy showing ulcer bleeding in the duodenal bulb after multiple previous clipping procedures.

A 39‐year‐old woman was admitted to the intensive care unit following multiple fractures and exploratory laparotomy after a traffic accident. She had no prior history of gastrointestinal disease. Upon admission, dark red fluid was observed in the gastric tube, and the occult blood test was positive (+). Laboratory tests showed a hemoglobin level of 6 g/dL and a hematocrit of 16%. Duodenoscopy revealed an actively bleeding ulcer at the junction of the duodenal bulb and descending part of the duodenum, classified as Forrest IIa. The patient was kept nil per os (NPO). Initial hemostasis was attempted using intragastric instillation of ice‐cold saline mixed with norepinephrine via a nasogastric tube. Somatostatin was administered to reduce splanchnic blood flow, and proton pump inhibitors (PPIs) were used to suppress gastric acid secretion and protect the gastric mucosa. Regular monitoring included hemoglobin levels, coagulation parameters, vital signs, and characteristics of nasogastric drainage. Component blood transfusions with packed red blood cells and fresh frozen plasma were administered to restore blood volume. Despite comprehensive conservative treatments, the patient's hemoglobin remained unstable and gradually declined. Dark red fluid continued to drain from the nasogastric tube. Endoscopic electrocoagulation and titanium clip placement were attempted on the first, third, and fifth days of hospitalization but failed to achieve lasting control of the bleeding (Figure 1). During this time, the patient underwent multiple transfusions and continued to receive medical hemostatic treatment. Hemoglobin levels declined gradually despite repeated exogenous supplementation (Tables S1–, S3), and dark red drainage persisted in the gastric tube.

### 2.1 Methods

**FIGURE 2:**
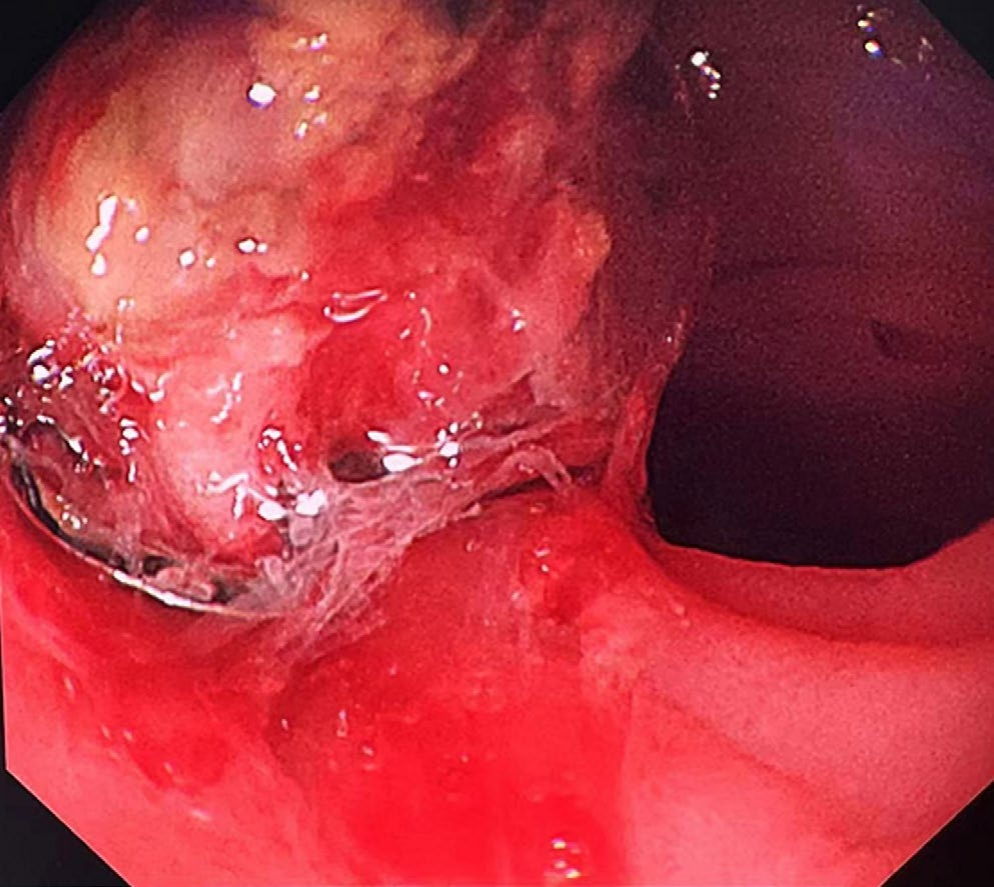
Endoscopic view post‐clipping. Successful hemostasis of the bleeding site using a ≥ 10 mm OTSC, followed by interventional embolization.

On the fifth day of hospitalization, a 10 mm OTSC (Micro‐Tech, Nanjing, China) was applied to the bleeding site. Although slight oozing remained after placement, the patient underwent selective embolization of the gastroduodenal and superior mesenteric artery branches. After embolization, bleeding ceased completely (Figure 2).

### 2.2 Outcome and Follow Up

**FIGURE 3:**
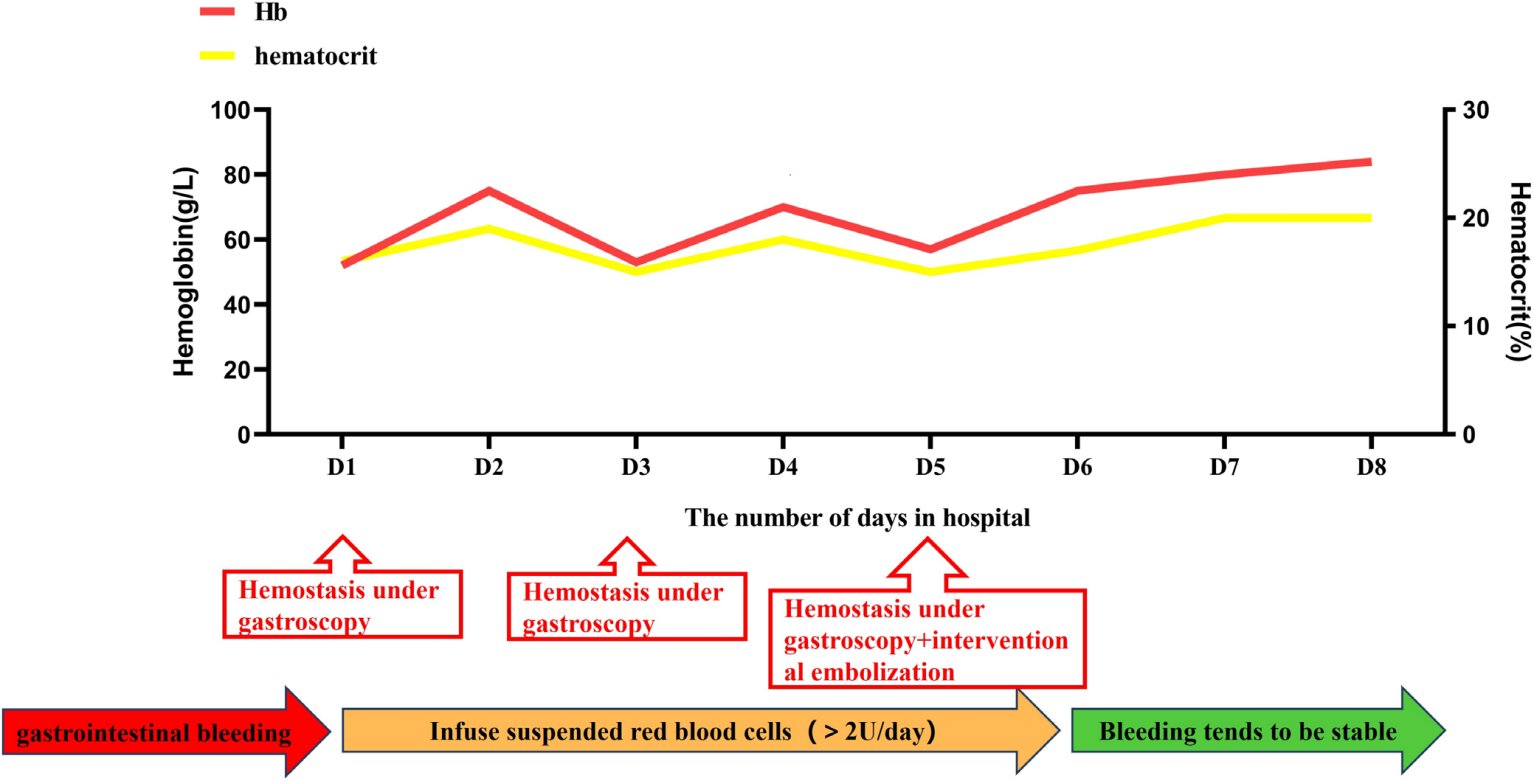
Treatment timeline for gastrointestinal bleeding. Days 1–3: Endoscopic attempts + other potential interventions. Day 5: OTSC + embolization.

After combined OTSC placement and embolization, the color and volume of gastric tube drainage gradually returned to normal. By the eighth day of hospitalization, the occult blood test was negative (−), and enteral nutrition support was initiated. Hemoglobin levels stabilized and rose without additional transfusions (Figure 3). The patient then began specialized treatment and rehabilitation. She was discharged after 26 days with no signs of gastrointestinal bleeding. At the one‐year follow‐up, she remained free of gastrointestinal symptoms.

## 3 Discussion

Post‐traumatic gastrointestinal ulcers are acute mucosal injuries caused by severe trauma or stress, leading to erosion or ulceration. In severe cases, complications such as bleeding or perforation may occur. With the widespread use of proton pump inhibitors and mucosal protective agents, along with an improved understanding of stress ulcers, the incidence of severe bleeding in these patients has decreased.

Multiple trauma often causes acute gastrointestinal mucosal erosion or ulcers, mainly presenting as upper gastrointestinal bleeding; perforation is rare. In some cases, bleeding may recur and become life‐threatening. Standard treatments include endoscopic electrocoagulation, tissue adhesives, and titanium clips. Stress ulcers can erode submucosal vessels, leading to serious hemorrhage. Prevention is the main strategy, but once bleeding occurs, endoscopic therapy is typically the first‐line treatment [6]. Surgical intervention is typically considered when endoscopic hemostasis fails, though its effectiveness remains uncertain and varies depending on the patient's overall condition and the location and size of the ulcer. In China, conventional treatments for stress ulcer bleeding include medications (such as proton pump inhibitors and H2 receptor antagonists), endoscopic techniques (electrocoagulation, tissue adhesives, titanium clips), and interventional radiology, all of which are generally effective [7, 8, 9]. Surgery may be an option if these methods fail, but it carries substantial risk and unpredictable outcomes. Recent reports in China have shown the successful use of OTSC as a standalone treatment for refractory gastrointestinal bleeding [10]. However, in challenging cases, especially duodenal bulb ulcers, persistent bleeding may continue despite medical and endoscopic approaches. Repeated clipping or electrocoagulation can increase the risk of perforation. When surgery is not feasible, and OTSC alone proves insufficient, combining it with interventional embolization may offer a safer and more effective alternative. In cases with complex vascular anatomy, OTSC may not fully block all bleeding vessels. Embolization enables targeted delivery of embolic agents to affected vessels and their branches. OTSC alone may be less effective for uncontrolled arterial bleeding due to continued high arterial pressure, while embolization can reduce this pressure and enhance bleeding control. The combined use of OTSC and embolization significantly improves the success rate and long‐term stability of hemostasis.

OTSC placement often fails to achieve full coverage for large or irregular bleeding lesions, while embolization can effectively target residual bleeding sites. In cases where bleeding is caused by tumor invasion of blood vessels, OTSC placement alone cannot address the underlying cause, while embolization not only controls bleeding but may also contribute to tumor suppression.

To prevent recurrence and improve long‐term outcomes, OTSC alone has limitations, as clip displacement or detachment can lead to rebleeding. Embolization reduces blood flow at the lesion, supports tissue healing, and lowers the risk of further bleeding, offering better long‐term stability. When surgery is contraindicated and OTSC is insufficient, embolization serves as a valuable adjunct. With the continuous advancement of medical technology, endoscopic hemostatic tools, including various clips, have significantly evolved [11]. The key challenge is selecting the most appropriate method for each clinical scenario. As treatment options increase, clinicians may adopt different strategies or combinations. However, choosing complex interventions with minimal patient benefit may be difficult to justify without high‐quality evidence.

Transcatheter arterial embolization (TAE) serves as an effective hemostatic option for gastrointestinal bleeding, particularly in patients who have failed endoscopic treatment or are unsuitable for endoscopic intervention. Potential complications include gastric or duodenal ischemia, rebleeding, infection, embolic material migration, renal impairment (such as contrast‐induced nephropathy), and vascular injury. Preoperative evaluation should include a thorough assessment of coagulation status, vascular integrity, and renal function to guide the selection of an appropriate embolization strategy. Postoperative monitoring is essential to detect complications such as abdominal pain, fever, or rebleeding and to ensure treatment safety and efficacy.

Previous studies have reported an overall adverse event rate of 2.1% for OTSC in gastrointestinal bleeding management, compared to 11.9% for interventional embolization [12, 13]. A retrospective single‐center study and systematic review indicated that there are risks of ischemia after interventional embolization and rebleeding after the use of OTSC. However, there was no statistical difference in the risks of ischemia and rebleeding between the two [9]. Some literature also suggested that the embolization group had a higher incidence of severe adverse events (12.9% vs. 1.5%) and in‐hospital mortality (22.6% vs. 9.1%), indicating that the long‐term risks of embolization were relatively high, while OTSC had a relatively greater advantage in this regard [7]. Therefore, we also recommend that in cases of endoscopic hemostasis failure or poor hemostasis effect, combined embolization therapy can be considered [14].

A review of six cases published on PubMed between 2019 and 2024 involving OTSC and/or embolization for gastrointestinal bleeding was conducted. Data on patient age, sex, bleeding cause, treatment method, and outcomes were summarized. Patients ranged from 3 to 94 years old. All achieved successful hemostasis after failure of conventional endoscopic treatments, with favorable survival outcomes in each case (Table 1).

**TABLE 1:** Summary of literature (2019–2024) on OTSC and interventional embolization for gastrointestinal bleeding.

Author	Age/gender	Causes of gastrointestinal bleeding	Treatment	Outcome
Huapei Song [15]	3/male	Severe burns	Gastroscopy combined with interventional embolization	Live
Megan Dunnigan [16] Willard	71/male	Complications of OTSC	Interventional embolization	Live
Jeffrey Abergel [17]	85/female	Multiple duodenal ulcers	Perform OTSC after the failure of interventional embolization	Live
	76/male	Severe burns	Perform OTSC after the failure of interventional embolization	Live
Yuhuang Guo [18]	82/male	After PCI	Interventional embolization	Live
Masashi Yamamoto [19]	94/male	Percutaneous endoscopic gastrostomy	Interventional embolization	Live

The current case describes a young female patient who developed an ulcer with bleeding at the junction of the duodenal bulb and descending colon following multiple severe injuries. Hemostasis was successfully achieved with combined OTSC and interventional embolization. However, comprehensive supportive care in the ICU, including large‐volume blood product transfusions, stabilization of the internal environment, and nutritional support, remained essential throughout the treatment. Therefore, as a single case report, broader validation through further studies is required to assess the generalizability and efficacy of this approach.

## 4 Conclusion

Case studies underscore the difficulties in controlling rebleeding resulting from post‐traumatic stress ulcers. When massive gastrointestinal bleeding cannot be controlled with conventional endoscopic techniques or when rebleeding occurs, combined OTSC and embolization may provide an alternative therapeutic approach.

## Author Contributions

**Qing Chen:** conceptualization, data curation, formal analysis, writing – original draft. **Shifeng Shao:** conceptualization, investigation, methodology, supervision, visualization, writing – review and editing.

## Ethics Statement

Medical research review by the Ethics Committee of the Army Medical Center of PLA. Written informed consent was obtained from the patient for the publication of all the images and data included in this article. Ethical review and approval were not required to publish the case details in accordance with the institutional requirements.

## Consent

The patient has consented to the submission of the case report to the journal.

## Conflicts of Interest

The authors declare no conflicts of interest.

## Supporting information


Table S1.

Table S2.

Table S3.

## Data Availability

All data generated or analyzed during this study are included in this published record [and its Additional files].
